# Correlation between psychological resilience and burnout syndrome in oncologists amid the Covid-19 pandemic

**DOI:** 10.1007/s00520-023-07660-3

**Published:** 2023-03-10

**Authors:** Anuska Budisavljevic, Renata Kelemenic-Drazin, Tajana Silovski, Stjepko Plestina, Natalija Dedic Plavetic

**Affiliations:** 1 Department of Medical Oncology and Hematology, General Hospital Pula, Pula, Croatia; 2 Department of Hematology, Oncology and Clinical Immunology, General Hospital Varazdin, Varazdin, Croatia; 3 Department of Oncology, University Hospital Centre Zagreb, Zagreb, Croatia

**Keywords:** Oncology, Burnout, Professional, Psychological resilience, Covid-19, Pandemic, Oldenburg Burnout Inventory, Brief Resilience Scale

## Abstract

**Purpose:**

Oncologists are predisposed to developing burnout syndrome. Like other health care professionals worldwide, oncologists have endured additional, extreme challenges during the Covid-19 pandemic. Psychological resilience presents a potential protective mechanism against burnout. This cross-sectional study examines whether psychological resilience eased burnout syndrome among Croatian oncologists during the pandemic.

**Methods:**

An anonymized self-reporting questionnaire was electronically distributed by the Croatian Society for Medical Oncology to 130 specialist and resident oncologists working in hospitals. Available for completion from September 6–24, 2021, the survey comprised demographic questions; the Oldenburg Burnout Inventory (OLBI), covering exhaustion and disengagement; and the Brief Resilience Scale (BRS). The response rate was 57.7%.

**Results:**

Burnout was moderate or high for 86% of respondents, while 77% had moderate or high psychological resilience. Psychological resilience was significantly negatively correlated with the OLBI exhaustion subscale (*r* =  − .54; *p* < 0.001) and the overall OLBI score (*r* =  − .46; *p* < 0.001). Scheffe’s post hoc test showed that oncologists with high resilience scored significantly lower on the overall OLBI (*M* = 2.89; SD = 0.487) compared to oncologists with low resilience (*M* = 2.52; SD = 0.493).

**Conclusion:**

The findings thus indicate that oncologists with high psychological resilience are at significantly lower risk of developing burnout syndrome. Accordingly, convenient measures to encourage psychological resilience in oncologists should be identified and implemented.

## Introduction

Physician burnout has received particular attention since it was proven to be correlated with treatment quality, patient satisfaction, and the overall performance of health care systems [1–3]. Oncologists are especially at risk of developing burnout. The main explanations are ever-growing demands for higher productivity, limited ability to control their own daily assignments, increased work overload with excessive administrative/electronic documentation, and the permanent imperative to track rapid scientific developments and keep pace with constant advances in oncological treatments [2–4].

During the last 10 years, 45–80% of oncologists worldwide experienced some of the symptoms associated with burnout [2, 4–8]. The term “burnout” was first defined in 1974 by Freudenberger, who used it to describe the emotional exhaustion experienced by public service workers unable to meet the demands and expectations of their environment [9]. Burnout syndrome often manifests through three basic symptoms: physical and emotional exhaustion, cynicism, and a feeling of inadequacy at work [10]. Demerouti et al. conceptualize burnout as comprising two dimensions: exhaustion and disengagement. Exhaustion is defined as the result of intense physical, emotional, and cognitive stress after prolonged exposure to specific demands; disengagement refers to distancing oneself from work and experiencing negative attitudes toward the object or content of work, a work task, or one’s job in general [11, 12].

The Covid-19 pandemic has led to profound changes in the organization of all levels of health care worldwide. It significantly affected health care for oncology patients [13, 14], with the oncological community promptly reacting to the pandemic by adjusting guidelines for cancer care [13, 14]. Oncologists had to quickly adopt new treatment guidelines and new ways of communicating with patients, such as telemedicine, virtual clinics, and virtual multidisciplinary teams. Also, the requirement to wear personal protective equipment (PPE) necessitated dramatic adjustments to standard forms of interpersonal communication with patients [13, 15, 16]. Despite the ongoing urge to provide the best possible health care for patients, oncologists had to consider all the potential risks of Covid-19 infection during the course of systemic therapy. These risks sometimes led to the adjustment or discontinuation of treatment, leaving oncologists deeply disappointed over the possible negative effects of these changes on treatment outcomes [14, 17, 18].

These adjustments to their daily working practices, along with several personal challenges (e.g., family stressors), have led to an increased sense of burnout among oncologists during the Covid-19 pandemic [18, 19]. The European Society for Medical Oncology (ESMO) Resilience Task Force found that the proportion of oncologists experiencing burnout rose from 25% at the onset of the pandemic (April 2020) to 57% after 10 months (February 2021) [20–22]. The same research emphasized the direct and very important consequences of burnout, including as many as 38% of oncologists considering a change of profession [20–22].

Psychological resilience has various definitions, but is mostly defined as an individual’s “ability to maintain or restore relatively stable psychological and physical functioning when faced with stressful life events and adversities” (Seiler, 2019) [23–26]. Research of psychological resilience began in the late 1980s when it was regarded as characteristic of extraordinary inner strength in exceptional individuals [23–25]. Over subsequent decades, the concept and understanding of psychological resilience have changed, and it is now perceived as a common phenomenon of adjustment when confronted with life stressors [23, 24, 26–28]. The nature of psychological resilience is very complex and multifactorial, comprising biological, psychological, social, and cultural factors that determine one’s adaptation and ability to recover quickly from adverse or stressful events [23, 29]. Psychological resilience also changes with age and depends on life circumstances [24, 30].

### The relationship between psychological resilience and burnout

Researchers have found that psychological resilience can potentially protect against burnout [2, 4, 5, 8]. Within the medical profession, physicians with higher psychological resilience report less likely to experience burnout symptoms [4]. Furthermore, psychological resilience is proven to mediate between burnout and mental health [4]. Although numerous articles have explored the relationship between psychological resilience and burnout in physicians, only a few have concentrated exclusively on oncologists.

Focusing on the Croatian health care system, this study has three objectives: (I) examine the impact of the Covid-19 pandemic on the everyday work of oncologists, including oncology education; (II) determine the prevalence of burnout and the level of psychological resilience in oncologists during the pandemic; and (III) examine the correlation between burnout and psychological resilience in the oncologist sample.

## Methods

A cross-sectional study was conducted during September 2021. An anonymous electronic survey was distributed by the Croatian Society for Medical Oncology to the e-mail addresses of 130 medical and clinical oncologists, all holding the position of either specialist or resident, and all working in general hospitals (GH) or university hospitals (UH) in the Republic of Croatia [31]. The Croatian-language survey was available for completion by respondents from September 6–24, 2021, and it comprised three main parts. The first part collected demographic data and posed questions about the impact of the pandemic on oncologists’ daily working practices. The second part assessed burnout using the Oldenburg Burnout Inventory (OLBI) [11], and the final part assessed psychological resilience using the Brief Resilience Scale (BRS) [32].

Since there is no data for burnout in the population of Croatian oncologist before the Covid-19 pandemic, burnout was also evaluated by one separate question answered “yes” or “no”: “Did you experience greater burnout (emotional and physical exhaustion and disengagement) during the Covid 19 pandemic compared to the pre-pandemic period?”.

Data on the medical facilities where respondents work were not collected so as to protect their anonymity, given that some facilities only employ one or two oncologists. The study was approved by the Institutional Ethics Committee Board of General Hospital Pula.

### Measuring instruments

Basic demographic data were collected on respondents’ age, gender (female/male), medical facility (GH/UH), position (specialist /resident), and length of service (≤ 10 years or > 10 years). We also asked whether geographical region where they worked had a high or low incidence of Covid-19. In addition, questions were posed about the use of telemedicine during the pandemic, the impact of PPE on communicating with patients, and the impact of the pandemic on oncology education, meaning whether they were able to maintain the requisite knowledge of developments in the field.

The OLBI was originally constructed to measure two dimensions of burnout: exhaustion and disengagement [11]. It is freely available for use in research, with no requirement for permission from the authors. The scale has previously been translated into Croatian and validated with a Croatian population [33]. In this study, the Cronbach’s *α* coefficients of reliability are satisfactory: 0.79 for the disengagement subscale, 0.85 for the exhaustion subscale, and 0.87 for the overall OLBI score.

The BRS is also free to use in research without express permission from the author [32]. Like the OLBI, it has previously been translated into Croatian and validated with a Croatian population [33]. In this study, the Cronbach’s *α* coefficient of reliability for the BRS is 0.81, which is in line with previous empirical findings [33].

### Statistical analyses

All statistical analyses were conducted using IBM SPSS Statistics for Windows version 26.0. Descriptive data are presented using arithmetic means, standard deviations, and percentages. To classify burnout and psychological resilience into three levels (“low,” “moderate,” and “high”), the respective scores were split into three groups: ≤ *M* − 1 SD; > *M* − 1 SD and < *M* + 1SD; ≥ *M* + 1SD. Differences between participant categories were examined using a *t*-test, setting 5% as the level constituting a statistically significant difference. We used Pearson correlation coefficients to indicate relationships between variables, again with 5% required to infer statistical significance.

A simple analysis of variance (ANOVA) was used to analyze differences in burnout in persons with low, moderate, and high levels of psychological resilience. We also used Scheffe’s post hoc test to further analyze the statistical significance of any group differences.

## Results

### Sociodemographic characteristics

Of the 130 contacted oncologists, 75 completed the questionnaire, representing a response rate of 57.7%. Respondents’ age ranged from 27 to 60 years (*M* = 41.66; SD = 9.23). Most participants were women (57.76%), and the majority of the sample were specialists (57.76%). Almost the same number of participants had up to 10 and 11 or more years of service (51% and 49%, respectively). Most respondents are employed in UH (48, 64%) (Table 1).

**Table 1:** Demographic characteristics of the sample of 75 oncologists

Characteristic	*n* (%)
Gender	
Female	57 (76.0)
Male	18 (24.0)
Position	
Resident	18 (23.9)
Specialist	57 (76.1)
Length of service	
≤ 10 years	38 (51.0)
> 10 years	37 (49.0)
Place of employment	69 (34.3)
General hospital	27 (37.3)
University hospital	48 (62.7)
Level of Covid-19 in oncologist’s workplace	
Low incidence	17 (22.7)
High incidence	58 (77.3)

### The impact of the Covid-19 pandemic on the daily work of oncologists

**Fig. 1:**
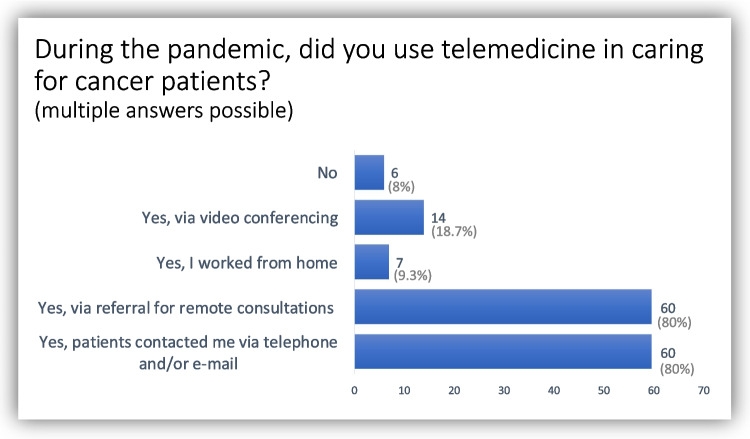
Use of telemedicine by oncologists during the Covid-19 pandemic

During the pandemic, almost all participating oncologists (92%) used some form of telemedicine while caring for cancer patients. Remote consultations and contacting patients via telephone or e-mail were most commonly reported (both 80%) (Fig. 1).

**Fig. 2:**
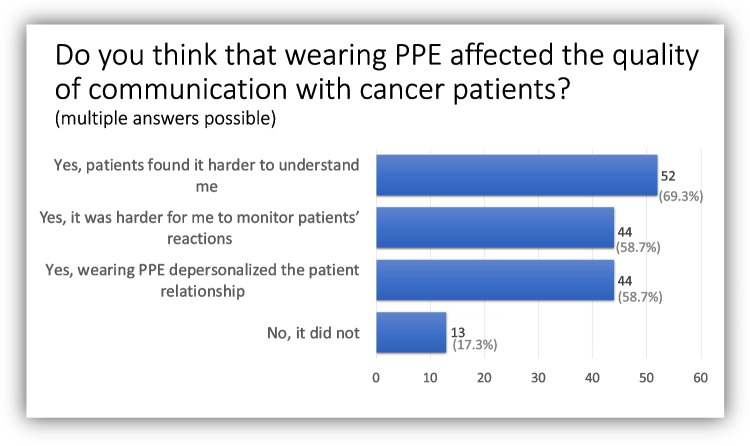
Impact of personal protective equipment on oncologists’ communication with patients

Overall, 83% of respondents reported that wearing PPE had affected the quality of communication with patients. More than two-thirds (69%) stated that their patients found it harder to understand them, while 58% felt that PPE depersonalized the patient relationship (Fig. 2).

**Fig. 3:**
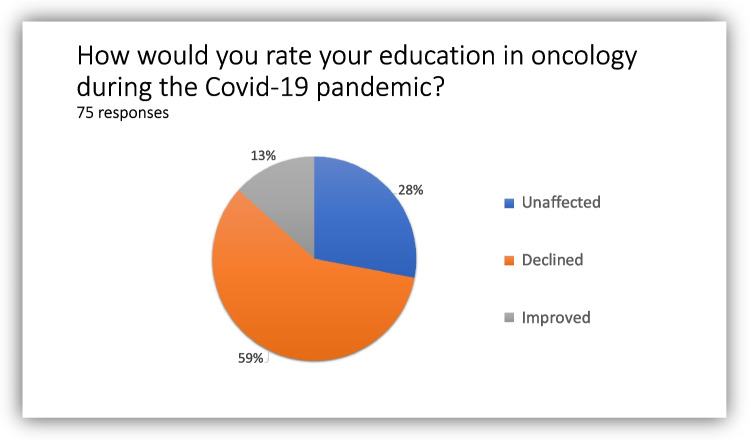
Impact of the Covid-19 pandemic on oncologists’ education

The majority of oncologists (58%) perceived they were not able to maintain the requisite knowledge of developments in the field during the Covid-19 pandemic, whereas only 13% reported an improvement (Fig. 3).

In response to the separate burnout question, the vast majority of oncologists (85%) answered that they did experience greater burnout during the pandemic than in the pre-pandemic period.

### Burnout and oncologists

Based on the overall OLBI score, respondents were divided into groups with low burnout (≤ 1.99), moderate burnout (2.0–3.11), and high burnout (≥ 3.11). Accordingly, 9 oncologists (12.3%) had low burnout, 50 (68.5%) had moderate burnout, and 14 (19.2%) had high burnout.

Burnout was not significantly correlated with age. Moreover, there were no statistically significant differences in burnout by gender, workplace (resident vs. specialist), service length (≤ 10 years vs. > 10 years), and Covid-19 level within their geographical region (low vs. high incidence).

UH oncologists scored significantly higher than GH oncologists on the overall OLBI (*p* = 0.046) and the disengagement subscale (*p* = 0.025). The *t*-test results are presented in Table 2.

**Table 2:** Results for the Oldenburg Burnout Inventory (OLBI) and Brief Resilience Scale (BRS)

		Mean (OLBI)	SD (OLBI)	*t*- test (sig.)	Mean (BRS)	SD (BRS)	*t*-test (sig.)
Gender	Male	2.46	0.642	− 1.606 (0.113)	3.28	0.783	0.048 (0.962)
	Female	2.73	0.605		3.27	0.694	
Workplace	Resident	2.54	0.580	− 0.070 (0.944)	3.34	0.692	0.448 (0.656)
	Specialist	2.55	0.553		3.26	0.721	
Length of service	≤ 10 years	2.52	0.575	− 0.396 (0.694)	3.28	0.697	0.081 (0.935)
	> 10 years	2.57	0.542		3.27	0.732	
Medical facility	General hospital	2.37	0.425	2.027 (0.046)	3.34	0.528	− 0.571 (0.570)
	University hospital	2.64	0.599		3.24	0.799	
Level of Covid-19 in oncologist’s workplace	Low incidence	2.55	0.543	− 0.044 (0.965)	3.33	0.771	− 0.370 (0.712)
	High incidence	2.55	0.565		3.26	0.697	

### Psychological resilience of oncologists

Based on the BRS total score, participants were divided into groups with low psychological resilience (≤ 2.57), moderate resilience (2.58–3.99), and high resilience (≥ 4.00). Accordingly, 17 participants (23.0%) had low resilience, 36 (48.6%) had moderate resilience, and 21 (28.4%) had high resilience.

Psychological resilience was not significantly correlated with age (*r* =  − 0.071; *p* > 0.05), and there were no statistically significant differences in resilience by gender, workplace, service length, medical facility, or Covid-19 incidence level. These results are reported in Table 2.

Psychological resilience has a statistically significant negative correlation with the OLBI exhaustion subscale score (*r* =  − 0.54; *p* < 0.001) and with the overall OLBI score (*r* =  − 0.46; *p* < 0.001). These results indicate that oncologists with higher psychological resilience had lower exhaustion and burnout. However, no statistically significant correlation was found between psychological resilience and the OLBI disengagement subscale (*r* =  − 0.28; ns).

#### Differences in the degree of exhaustion, disengagement, and burnout by resilience level

The ANOVA results show that oncologists with different levels of psychological resilience have statistically significant differences in the level of exhaustion (*F* (2.69) = 9.46, *p* < 0.001). In Scheffe’s post hoc test, oncologists with high psychological resilience had significantly lower exhaustion (*M* = 2.32; SD = 0.625) than oncologists with moderate resilience (*M* = 2.67; SD = 0.562) and low resilience (*M* = 2.62; SD = 0.562) = 3.13; SD = 0.459). Again, however, oncologists with different levels of psychological resilience do not significantly differ with respect to the level of disengagement.

The ANOVA results also show that oncologists with different levels of psychological resilience significantly differ in the overall OLBI score (*F* (2.69) = 5.07, *p* < 0.01). In Scheffe’s post hoc test, oncologists with high psychological resilience achieved a significantly lower overall OLBI score (*M* = 2.89; SD = 0.487) compared to oncologists with low resilience (*M* = 2.52; SD = 0.493). These results are reported in Table 3.

**Table 3:** Results of ANOVA test

	Resilience	Mean	SD	*F* (*df*)	Sig
Exhaustion	Low	3.13	0.459	9.458 (2/69)	*p* < 0.001
	Moderate	2.67	0.562		
	High	2.32	0.625		
Disengagement	Low	2.66	0.627	1.519 (2/70)	*p* > 0.05
	Moderate	2.36	0.548		
	High	2.36	0.669		
Overall burnout	Low	2.89	0.487	5.070 (2/69)	*p* < 0.01
	Moderate	2.52	0.493		
	High	2.34	0.614		

## Discussion

The importance of preserving well-being and avoiding burnout in physicians, as well as the consequent impacts on health care quality, have become increasingly recognized in recent years. The Covid-19 pandemic has further strained health care systems and professionals around the world, who, in addition to caring for patients, also faced concerns over their own safety.

Croatian oncologists very quickly adapted to the new requirements and standards in caring for cancer patients, and the vast majority of surveyed oncologists (86%) reported using telemedicine during the pandemic. This rate exceeds that reported by ESMO, who found in 2020 that 49% of oncologists had engaged in remote consultations during the pandemic [20].

Participating oncologists reported that wearing PPE significantly interfered with their interaction and communication with patients. Nearly two-thirds of oncologists felt that wearing face masks made it harder to monitor patients’ reactions and felt that this depersonalized patient relationships. Around 55% of communication is nonverbal, with facial expressions contributing significantly [15]. Positive facial expressions by the doctor play a key role in calming the patient. Consequently, where the doctor and patient must both wear masks, communication becomes much more difficult [15]. Medical staff associate difficult communication during the Covid-19 pandemic with negative emotions [34]. Quantitative studies emphasize that the quality of patient relationships is important for finding meaning in one’s work, leading to reduced risk of burnout [8]. The impact of wearing of PPE on the quality of doctor–patient relationships deserves further research.

Despite numerous educational lectures being made available online, two-thirds of oncologists believed their education declined during the pandemic. Earlier studies revealed that worrying about education and career is significantly associated with developing burnout, both during and before the Covid-19 pandemic [4, 20].

As many as 85% of oncologists affirmed that they had experienced greater burnout during, compared to before the pandemic, while 86% showed moderate to high levels of burnout on the OLBI. In a pre-pandemic study of burnout among oncologists in Eastern Europe during 2019, Kust et al. found that 71% of the sample (including Croatian oncologists) experienced high burnout [35]. Although Kust et al. used a different method to us (the Maslach Burnout Inventory), both studies find a high percentage of oncologists with burnout symptoms and an increased level of burnout in Croatian oncologists during the pandemic. Various other studies have also reported rising levels of burnout among doctors during the pandemic [17–21, 36, 37,38].

Previous studies have demonstrated that gender and work experience are each correlated with burnout: specifically, both female gender physicians and physicians with a shorter time in service have been found more likely to experience burnout [20, 35, 36]. However, our research found no statistically significant differences in burnout by gender or time in service. These contradictory findings may be explained by the relatively small number of respondents, especially the proportion of resident oncologists, who generally have shorter time in service. Considering the workplace, we find UH oncologists to be at higher risk of burnout and of disengagement compared to GH oncologists, but observe no difference between them regarding the risk of exhaustion. These results are consistent with previous findings that employment in an UH poses a high risk of developing burnout [36].

Doctors’ psychological resilience has been the focus of numerous studies in recent years, especially those exploring psychological resilience as a protective factor against burnout [2, 4, 5, 8]. The very nature of psychological resilience is complex and multifactorial [2, 24], affected by individual personality, social support, personal interests outside work, and the ability to overcome past adversities in life [37]. According to previous research, physicians generally have high psychological resilience [2, 8, 37, 39]. Moreover, West et al. found in the USA that the level of psychological resilience is higher among physicians than in the overall working population [8]. In line with these findings, 77% of participating oncologists showed high or moderate psychological resilience.

We find that psychological resilience is strongly negatively correlated with burnout and the exhaustion subscale but not with the disengagement subscale. Our results also show that the level of psychological resilience plays an important role when facing burnout: Croatian oncologists with high psychological resilience are significantly more likely to have lower burnout when compared with oncologist with low psychological resilience. Moreover, oncologists with high psychological resilience are at significantly lower risk of developing emotional and physical exhaustion, comparing with oncologist with low resilience levels, yet disengagement is not correlated with the level of psychological resilience. West et al. reported that 30% of physicians with high psychological resilience nonetheless experienced high burnout [8], which highlights the need to deepen understanding of the relationship between psychological resilience and burnout by considering potential mediating variables in future studies.

### Limitations and future research directions

As this study has several limitations, the results should be interpreted with caution. First, collecting data online leads to self-selection of participants that may affect the findings. However, during the Covid-19 pandemic, online data collection proved an extremely useful method, and given the wide geographical distribution of potential respondents, in-person data collection would have significantly complicated data collection and reduced the number of participants.

Second, this study had a relatively small number of participants. However, this largely reflects the chosen context: in 2020, a total of 79 medical oncologists and 132 clinical oncologists were registered in Croatia [40]. Only oncologists employed in GH or UH were included in the study, giving a target population of 130 oncologists, 75 of whom ultimately participated were contacted and a response rate of 57.7% was achieved. Another issue is the high proportion of female oncologists in our sample (76%). Although data from 2020 show that 63.4% of employed medical doctors in Croatia were women [40], it is still not possible to generalize our results to Croatia’s entire population of oncologists.

Regarding the impact of the Covid-19 pandemic on burnout, one should bear in mind that our study is cross-sectional and so reports information on the level of oncologists’ burnout only during the pandemic. Since no prior study has explored the level of burnout among only Croatian oncologists, we cannot draw conclusions on whether the pandemic led to higher burnout, though the question asking respondents to compare burnout before and during the pandemic suggests Covid-19 pandemic did lead to increase in burnout levels. For this reason, we plan to conduct another study measuring burnout and psychological resilience among Croatian oncologists after the pandemic, seeking to discern the pandemic’s impact on burnout.

Psychological resilience is the central focus of numerous studies. The concept is difficult to define because of its complex nature, with social, psychological, biological, and cultural factors all interacting to determine how an individual responds to stress [2, 8, 23, 24, 37]. Therefore, psychological resilience should be seen as not only an intrinsic trait but also a skill to be learned and mastered [24]. Considering the relationship between psychological resilience and burnout, it is clearly necessary to define the modifiable factors of psychological resilience. Prospective longitudinal studies are needed to research the dynamics of the psychological resilience–burnout relationship while also exploring interventions to act on the modifiable factors and thereby strengthen psychological resilience. For example, self-compassion and mindfulness are often integral parts of psychological resilience that can potentially be learned or strengthened [4, 27, 30].

## Conclusion

In health care systems, care for doctors should have equivalent priority to the traditional main concern of patient care. Health care organizations should work systematically on developing positive health policies, focused on individuals in the health care system and on organizational problems, with the aim of promoting physicians’ well-being [2, 4]. New and emerging therapeutic options for cancer patients are constantly on the horizon, promising more efficient treatment outcomes and longer treatments for metastatic cancer patients. However, these developments also increase the workload of oncologists, raising their risk of burnout. It is well known that burnout symptoms can be reduced by improving working conditions through occupational alignment [2, 4]. Moreover, the absence or lower levels of burnout lead to more productive doctors, more satisfied patients, and better treatment outcomes [1, 7]. Finally, given the clear negative correlation between psychological resilience and burnout, interventions that strengthen psychological resilience in oncologists should be systematically identified and implemented.

## Data Availability

The data presented in this study are available on request from the corresponding author.
